# Preparation of PLA Nanoparticles and Study of Their Influencing Factors

**DOI:** 10.3390/molecules29235566

**Published:** 2024-11-25

**Authors:** Xinyu Zhang, Qing Luo, Fengying Zhang, Xinye Zhao, Ying Li, Ning Yang, Liangshan Feng

**Affiliations:** 1 Key Laboratory of Regional Environment and Eco-Remediation of Ministry of Education, Shenyang University, Shenyang 110044, China; zhangxinyu147147@163.com (X.Z.); zhaoxinye10@163.com (X.Z.); lily_01333@163.com (Y.L.); 2 Shenyang Institute of Science and Technology, Shengyang 110167, China; meiying851745389@163.com; 3 Liaoning Academy of Agricultural Sciences, Shenyang 110161, China; fenglsh@163.com; 4 Plant Protection College, Shenyang Agricultural University, Shenyang 110866, China; yangninglaas@126.com

**Keywords:** polylactic acid nanoparticles, emulsification-solvent volatilization, response surface methodology, polydispersity index

## Abstract

Nanoparticles (NPs) have attractive properties that have received impressive consideration in the last few decades. Polylactic acid nanoparticles (PLA-NPs) stand out as a biodegradable polyester with excellent biocompatibility. This investigation introduces PLA-NPs prepared by using the emulsification-solvent volatilization (O/W) method. The effects of ultrasonication time, organic-to-aqueous phase volume ratio, surfactant Tween-20, and PLA on particle size as well as the polydispersity index (PDI) were investigated using a one-factor combination with Response Surface Methodology (RSM). The result indicates that, on the one hand, PLA was the key factor affecting particle size, which gradually increased as the amount of PLA increased from 0.01 to 0.1 g. The particle size of NPs gradually decreased as the surfactant Tween-20 increased from 0.25 mL to 1 mL in the aqueous phase. The volume ratio of the organic phase to the aqueous phase increased from 1:10 to 1:1, with the particle size initially decreasing (from 1:10 to 1:5) and subsequently increasing (from 1:5 to 1:1). As the ultrasonication time increased from 20 min to 40 min, the particle size initially increased (from 25 to 30 min) and then decreased (from 30 to 40 min). On the other hand, Tween-20 was the main factor of PDI, and with the increase of Tween-20, PDI changed significantly and increased rapidly. The volume ratio of the organic phase to the aqueous phase increased from 1:10 to 1:1, resulting in the stabilization and subsequent gradual decrease of the PDI. With the increase of ultrasonication time (20–40 min), PDI tended to be stable after the increase. The effect of PLA on PDI was not significant, and the change of PLA concentration did not cause a significant change in the size of PDI.

## 1. Introduction

Nanotechnology, which explores the properties and applications of materials at the nanoscale, is rapidly advancing and progressively permeating various fields including the chemical industry, medicine, energy, life sciences, and agriculture, thereby offering a novel scientific approach to traditional disciplines [1,2,3]. The development of biodegradable, eco-friendly, and renewable nanomaterials had been prompted by China’s growing focus on environmental protection in recent years [4,5]. Polylactic acid (PLA) stands out as a biodegradable polyester with excellent biocompatibility [6]. PLA has excellent mechanical, physical, structural and thermal properties that make it suitable for a variety of applications [7]. However, PLA has limitations such as low impact toughness, hydrophobicity and slow degradation at ambient temperatures. PLA has a long history of use in biomedical applications, with the U.S. Food and Drug Administration (FDA) approving its use as early as 1970 [8]. Since its approval by the U.S. Food and Drug Administration (FDA) for use in humans [9], PLA has been used primarily for wound management, orthopedic and fixation devices, drug delivery, and tissue engineering. PLA-NPs can encapsulate drug molecules in their nanoparticles, preserving the activity of the drug while providing a slow release effect [10]. Shuqiang Liu et al. used two biopolymers, polyglycolactone (PGA) and polycaprolactone (PCL), as carriers for ciprofloxacin (CPFX), and coated CPFX-PCL/PGA onto PLA sutures. The material developed has suitable strength, antimicrobial capacity, and biodegradability suitable for tendon suturing (https://www.researchgate.net/publication/301826141_Induction_of_P-glycoprotein_expression_and_activity_by_Aconitum_alkaloids_Implication_for_clinical_drug-drug_interactions, accessed on 18 October 2024). Sun et al. prepared PLA-pyrimidinol nanomicrospheres using a single-factor approach, revealing that surfactant concentration significantly influenced particle size [10]. The double emulsion method (W/O/W) allows the formation of nanocapsules in which the aqueous core accommodates the hydrophilic drug and the polymer shell accommodates the hydrophobic drug [11,12]. However, due to the presence of biological barriers in the human body, routes of administration and drug targets limit the therapeutic effects of polymeric nanoparticles (PNPs), with the main limiting factor being particle size. Danaei et al. found that PLA-coated drug capsules smaller than 50 nm can be used in renal therapy, particles of 100–150 nm can penetrate deep into the body’s capillaries, and PLA capsules larger than 200 nm can also assist phagocytosis in the treatment of pathogens [13]. Shao et al. developed poly(lactic acid) microspheres containing retinoic acid with smooth morphology, good dispersion, and a particle size of 1.3 μm [14]. They achieved them by varying the volume ratio of inner and outer aqueous phases, adjusting stirring speed shear force, and employing gelatin and Tween-20 as emulsifiers in the outer aqueous phase. However, this approach considerably overlooked the impact of PLA itself on particle size and the preparation of micron-sized particles was far less widely used than nano-sized particles. While these studies laid a scientific foundation for understanding nanoparticle preparation and its influencing factors, they also underscored the need for more comprehensive investigation. Hence, this study aimed to systematically study the optimal preparation of PLA-NPs and the factors influencing their formation. 

The emulsion was prepared using the O/W method, with the aqueous solution as the external phase. The low-toxic, biodegradable surfactant Tween-20 was selected as the emulsifier for the experiment [15]. Tween-20, a surfactant with a large hydrophilic–lipophilic balance, can be added to increase the dispersion of the organic phase in the aqueous phase, making the system more stable overall [16]; for this reason, it was selected as the emulsifier for the experiment [10]. The solvents of the inner phase include acetone, dichloromethane (DMC), ethyl acetate, and so on, of which DMC was slightly soluble in water and had better solubility for PLA, thus it was adopted [17]. 

In this study, PLA-NPs were prepared, and the factors affecting the particle size and PDI of PLA-NPs were examined through a combination of the one-factor control variable method and application response surface, with a view to providing technical and parametric support for the preparation of environmentally friendly PLA-NPs.

## 2. Results and Discussion

### 2.1. Optimization of One-Factor Conditions

#### 2.1.1. Ultrasonication Time

**Figure 1:**
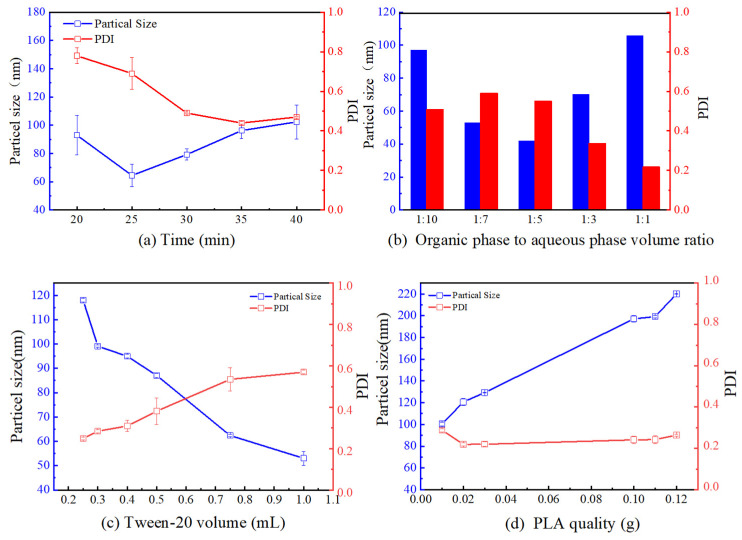
Optimization of PLA-NPs and PDI one-factor conditions.

When ultrasonic waves are transmitted through a vortex into an emulsion formed by blending an organic phase with an aqueous phase, this process includes the phenomena of agglomeration and dispersion of microbubbles in the medium [18]. This brief ultrasonic transmission process generates heat, leading to a reduction in the size of microbubbles in emulsion that was not uniformly mixed. Through continuous ultrasound, strong turbulence of the droplets is induced, resulting in microbubble dispersion [8]. Increasing the ultrasonication time extends the contact time between the emulsifying medium and the shear stress provided by the sonication process. As depicted in Figure 1a, from 20 to 25 min, increasing ultrasound time decreased particle size, a trend similar to that observed by Ruiz Esneyder et al. [19]. The reduction in particle size was mainly due to the dispersion of organic phase droplets that did not aggregate in the homogeneous aqueous phase [20]. However, when the ultrasound time was extended to 30, 35, or 40 min, particle size increased. This was attributed to excessive sonication time, leading to elevated solution temperatures and accelerated organic solvent volatilization rates. The rapid precipitation of PLA caused by the accelerated volatilization of organic solvents led to the appearance of a larger number of particles at the same time. This particulate matter underwent the phenomenon of agglomeration at high temperatures, and the particle size became larger [11]. PDI functions as a metric to describe the level of heterogeneity present in the particle size dispersion of colloidal systems [12]. PDI is evaluated by squaring the quotient of the particle size distribution’s standard deviation to its mean [13]. In samples where the PDI is zero, it indicates complete homogeneity. When PDI exceeds 0.7, it indicates a broader distribution of particle sizes. As is evident from Figure 1a, when the ultrasonication time was from 20 to 25 min, the PDI was notably high, indicating unsuitability for DLS measurements. As depicted in Figure 1a, when ultrasonication time was greater than or equal to 30 min, the average particle size error was relatively small, and the aqueous and organic phases exhibited a mixed uniform state, with a stable trend in PDI variation. Hence, considering time and cost efficiency, ultrasonication time of 30 min was chosen as the one-factor optimization condition.

#### 2.1.2. Volume Ratio of Organic Phase to Aqueous Phase

As can be seen in Figure 1b, the particle size of PLA-NPs showed a decrease followed by an increase as the volume ratio of the organic phase to the aqueous phase increased. Firstly, when the volume ratio increased from 1:10 to 1:5, the particle size decreased while the PDI remained relatively stable. This phenomenon could be attributed to the viscosity of the emulsion, which was influenced by the organic phase content. An increased proportion of the organic phase leads to elevated emulsion viscosity, necessitating greater external force for emulsification and dispersion [21]. Therefore, under the same ultrasonic conditions, the particle size was not easily dispersed, with the particle size becoming smaller. When the ratio of the organic phase to the aqueous phase volume was elevated from 1:5 to 1:1, the particle size of PLA-NPs increased, but PDI decreased. The main reason for the increase in particle size was that the total volume of the reaction system remained unchanged. The closer the volumes of the aqueous and organic phases are within the same volume, the better the compatibility of the aqueous phase with the organic phase, and the less likely it was to be dispersed. Generally, as the volume ratio of the organic phase to the aqueous phase decreases, the concentration of the emulsifier increases, resulting in a better spheroidization effect [22]. Consequently, the PDI gradually decreases and eventually stabilizes. As shown in Figure 1b, when the volume ratio ranged from 1:7 to 1:5, the error was minimal. At this point, the particle size remained stable, and the PDI stabilized within a reasonable range. This indicated that the system was more suitable for the preparation of NPs.

#### 2.1.3. Surfactant Tween-20

As can be seen in Figure 1c, it was evident that as the amount of surfactant in the aqueous phase increased from 0.25 mL to 1 mL, the particle size of PLA-NPs became gradually smaller and the PDI became lower. This occurred due to the rise in surfactant concentration within the aqueous solution, resulting in a reduction of its surface tension [23]. Consequently, it enhanced the dispersion of the organic phase within the aqueous phase and facilitated the creation of a hydrophilic film at the interface between the organic and aqueous phases. This film acted as a barrier, preventing nanoparticle agglomeration and increasing the stability of the system. However, PDI increased rapidly as the amount of surfactant approached 1 mL. This was due to the fact that too high a concentration of surfactant increased the hydrophilicity, which could result in the formation of rod-like or even multilayered structures in the aqueous phase, negatively affecting the formation of NPs [24]. Therefore, NPs could be uniformly dispersed in the system only if the surfactant was in the appropriate concentration range. As is evident from Figure 1c, when Tween-20 was in the range of 0.3–0.4 mL, the size of the prepared PLA-NPs was in the range of 100 nm and the PDI was small, indicating that the system was suitable for the synthesis of NPs.

#### 2.1.4. PLA

As shown in Figure 1d, PLA increased from 0.01 g to 0.12 g. The NPs size increased linearly. This was attributed to the fact that when the PLA was too low (less than 0.01 g), microbubbles formed during particle formation, causing collapse during the curing process, resulting in poor shaping. However, as the PLA increased, the viscosity of the organic phase increased. Under the same ultrasonic conditions, it became less easily dispersed, leading to an increase in the nanoparticle size [25]. During testing, PDI of PLA-NPs remained stable. From Figure 1d, it can be observed that PLA ranged from 0.1 g to 0.12 g, with an average particle size of 200 nm. At this point, the preparation method demonstrated better dispersion and stability.

### 2.2. RSM for the Preparation of PLA-NPs and the Factors Affecting Them

**Figure 2:**
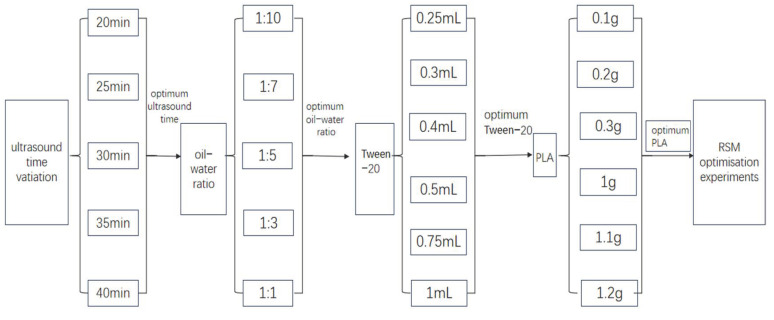
A diagram of the protocol carried out step by step.

A diagram of the single-factor optimization step-by-step protocol is shown in Figure 2. A total of 17 experiments were carried out under two response surfaces and three factors. In the one-factor experiment, it was found that although the particle size increased slightly at a sonication time of 30 min relative to 25 min of sonication, both the standard deviation of the particle size and PDI of the particles decreased significantly. As the ultrasound time increased, the size of the particles gradually increased and the PDI index also tended to stabilize. In order to obtain small-sized PLA-NPs, the ultrasound time was kept at the same 30-min level as in RSM. This research methodology was set up to optimize the method of preparing PLA-NPs and to determine the effect of the factors on the particle size and the PDI. The three factors, namely the volume ratio of the organic phase to the aqueous phase, Tween-20 (mL), and PLA (g), were assigned with their highest and lowest values. For the above 17 sets of experiments, the RSM of Design-Expert-11 software was used, and the results of the analyses of each observed response variable were tested using the analysis of variance (ANOVA) of the response surface quadratic model [26]. Before performing the ANOVA, it was determined that the data were normally distributed. The values of the statistical parameters for PLA-NPs and PDI as a response surface are shown in Table 1 and Table 2: Degrees of Freedom (DF) and the sum of squares (SS), respectively, Mean Square (MS), Multiple Correlation Coefficient (R^2^), Adjusted R^2^, Standard Deviation, F value, *p*-value.

#### 2.2.1. Effect of Factors on Particle Size of PLA-NPs

ANOVA for the quadratic regression model, presented in Table 2, revealed that the F-value of 14.89 demonstrated the significance of the model, and the probability of such a large F-value being due to the effect of error was only 0.09%. When the *p*-value was below 0.05, it suggested significance for the model term, whereas if the *p*-value exceeded 0.1, it implied insignificance for the model. The *p*-value of the three factors selected in this experiment was under 0.05, indicating that the change of particle size was related to factors A (organic phase to aqueous phase volume), B (Tween-20), and C (PLA). This further demonstrated that the selection of the conditions for the one-factor experiments was correct. In particular, factor C was extremely significant (*p* < 0.0001), indicating that the amount of PLA had the main effect on the particle size. This was consistent with the results of the one-factor experiments, suggesting that adjusting the concentration of PLA made it easier to prepare nanoparticles with different sizes. As can be seen from Table 2, based on the RSM, it can be stated that the AB, AC and BC models were not significant, indicating that there is no significant difference between the two-by-two interactions on the size of the synthetic particle size, suggesting that we can rely on the one-factor optimization conditions as a reference. The correlation coefficient (R^2^) was 0.9503, meaning that the model accounted for 95.03% of the variability in particle size. The adjusted R^2^ value was similarly elevated, standing at 88.65%. Equation (1) of the quadratic regression model with particle size as the objective function was obtained according to the software analysis.

(1) $$Particle\;size=123.3+57.51\;A-63.34\;B+1164.32\;C+13.73\;AB\;\;+330.49\;AC-52.89\;BC-50.16\;A^{2}-4.74\;B^{2}-4689.75\;C²$$

**Figure 3:**
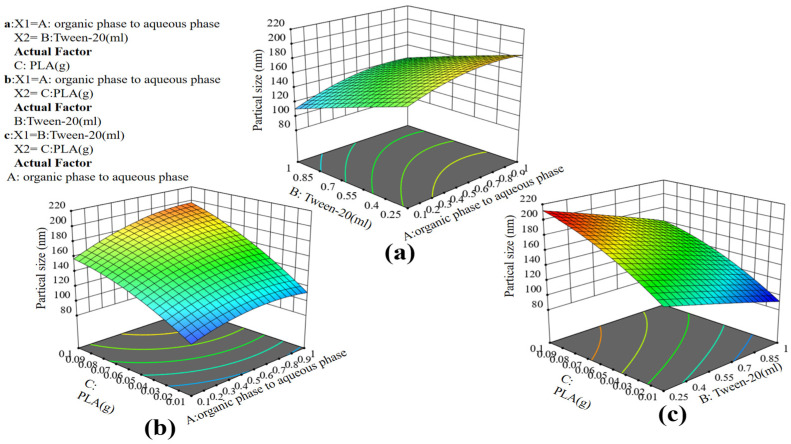
RSM (**a**) shows the relationship between average particle size and PLA, (**b**) Tween-20, (**c**) organic phase to aqueous phase volume.

From the overall view shown in Figure 3, it can be observed that factor A (organic phase aqueous phase volume ratio) and factor B (Tween-20) were negatively correlated with particle size, and factor C (PLA) was positively correlated with particle size, so particle size decreases with the increase of organic phase aqueous phase volume ratio and Tween-20. As the amount of PLA increases, the particle size gradually increases. In Figure 3a, it is evident that different volume ratios of the organic phase to the aqueous phase had a significant impact on the particle size of the prepared nanoparticles. Nanoparticles with a small size (100 nm) could be obtained when the difference between the two volume ratios was large and, at the same time, Tween-20 reached its maximum value. As shown in Figure 3b, the particle size increased with the increase of PLA, and when the particle size of PLA-NPs was around 200 nm, the amount of PLA required to reach the maximum value was 0.1 g, and at this time, the organic phase to aqueous phase volume was at a minimum of 1:1. As shown in Figure 3c, increasing Tween-20 led to a decrease in particle size until it reached a minimum value, requiring a Tween-20 of 1 mL and a minimum PLA of 0.01 g to achieve this. In summary, an increase in the volume ratio of the organic phase to the aqueous phase and Tween-20, and a decrease in PLA, would result in a smaller particle size. This conclusion was consistent with the findings obtained in the one-factor experiments. Stable nanoparticles with particle sizes ranging from 96.51 nm to 207.16 nm could be obtained through modeling.

#### 2.2.2. Effect of Factors on PDI of PLA-NPs

**Figure 4:**
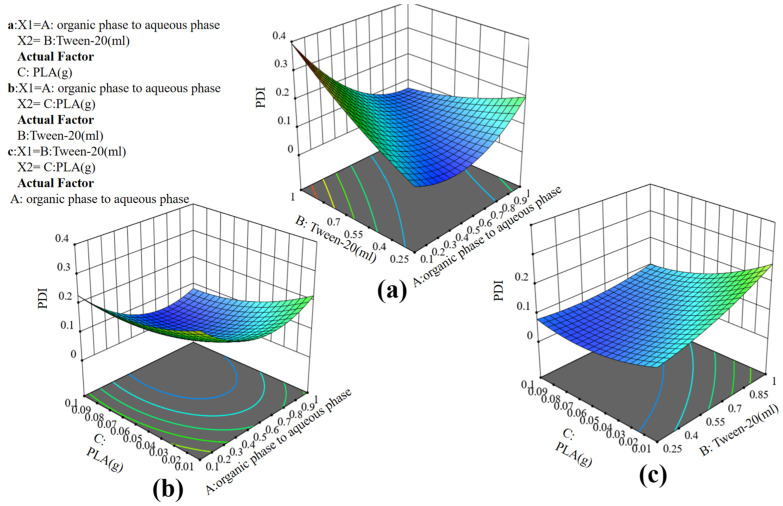
RSM (**a**) relationship between PDI and PLA, (**b**) relationship between PDI and Tween-20, (**c**) relationship between PDI and organic phase to aqueous phase volume.

The analysis of variance of the quadratic regression model shown in Table 3 indicated that the F value of 11.07 highlighted the importance of the model, with the probability of such a large F value being due to the effect of error only 0.23. When the *p*-value is below 0.05, it indicates that the model term is significant, and when the *p*-value exceeds 0.1, it indicates that it is not significant [27]. Moreover, when the *p*-value was less than 0.001, it denoted extreme significance. Under these circumstances, factors A, B, C, AB, and A^2^ were all significant with *p*-value less than 0.05, suggesting a correlation between the change in PDI stability and factors A, B, and C. This also affirmed the correctness of selecting the one-factor experimental conditions. Notably, the effects of factors A and B on PDI stability were extremely significant (*p* < 0.0001), and their interaction (AB) was also extremely significant, highlighting the importance of organic phase to aqueous phase volume and Tween-20 to PDI stability. As can be seen in Figure 4a, it is evident that an increase in organic phase to aqueous phase volume and Tween-20 led to a continuous increase in PDI, consistent with the results of the one-factor experiments. The influence of factor C (PLA) on the multiexponential coefficient was found to be insignificant. The correlation coefficient (R^2^) of the model stood at 0.9503, suggesting that the model accounted for 95.03% of the variation in particle size. The adjusted R^2^ was similarly elevated, reaching 88.65%. The model was analyzed using software to derive Equation (2) of the quadratic regression model with particle size as the objective function.

(2) $$PDI=123.33+57.51\;A-63.34\;B+1164.32\;C+13.73\;A+330.49\;A-52.89\;B-50.15\;A^{2}-4.74\;B^{2}-4689.75\;C^{2}$$

In Figure 4a, it can be observed that the three-dimensional image formed by factors A and B displays protrusions on the sides and concavity in the middle, indicating their significant impact on PDI, with their interaction also having a notable effect. The optimal PDI value was expected to appear between the two factors. Overall, factor A (organic phase to aqueous phase volume) and factor B (Tween-20) were positively correlated with PDI, suggesting that maintaining stability required selecting intermediate values for the organic phase, aqueous phase, and Tween-20. From Figure 4b, the three-dimensional image appeared relatively flat, indicating that factor C (PLA) had an insignificant effect on PDI size. In Figure 4c, while both factors A and B were significant, factor B was more significant than factor A, suggesting that Tween-20 significantly influenced PDI in NPs preparation.

### 2.3. Optimizing Results

RSM, a type of multivariate quadratic regression technique, addresses the limitations of dealing exclusively with discrete level values [28]. This method employs polynomial functions to model the relationships between the influencing factors and the observed indicators in multifactorial experiments, analyzing factor interactions with the goal of comprehensively assessing the selected parameters [29]. RSM boasts advantages such as requiring fewer tests, shorter durations, lower costs, and yielding high precision regression equations [30]. It has achieved notable success in domestic research. In this experiment, nanoparticle diameters ranging from 97 to 207 nm were achieved using RSM, with PDI stability between 0.09 and 0.35. Four factors influencing particle size and PDI were determined through one-factor testing. The experimental results identified the three most significant influencing factors, which exhibited consistent effects on both particle size and PDI during the validation of RSM. The application of RSM highlighted PLA as the primary factor affecting particle size, aligning with the findings of the one-factor experiments. This suggests that adjusting PLA facilitates the preparation of NPs with diverse sizes, while PLA has minimal impact on PDI. In the one-factor experiments as well as RSM, it could be seen that PDI was stable at around 0.2. Utilizing RSM revealed that factor B (Tween-20) was the primary factor influencing PDI size. Strictly controlling the amount of Tween-20 was a decisive factor in determining whether or not we were able to successfully create synthetic PLA-NPs.

### 2.4. Surface Morphology by SEM and Size by DLS

The suspension obtained in the above was washed repeatedly with anhydrous ethanol and deionized water, centrifuged three times, then washed again, and finally PLA was obtained by the freeze-drying method. A certain amount of dried PLA powder with different particle sizes was taken out and uniformly coated on a conductive adhesive. After gold spraying, the particle size and surface morphology of PLA were observed using a Hitachi S-4800 cold field emission scanning electron microscope at a voltage of 5 kV (Hitachi, Tokyo, Japan).

**Figure 5:**
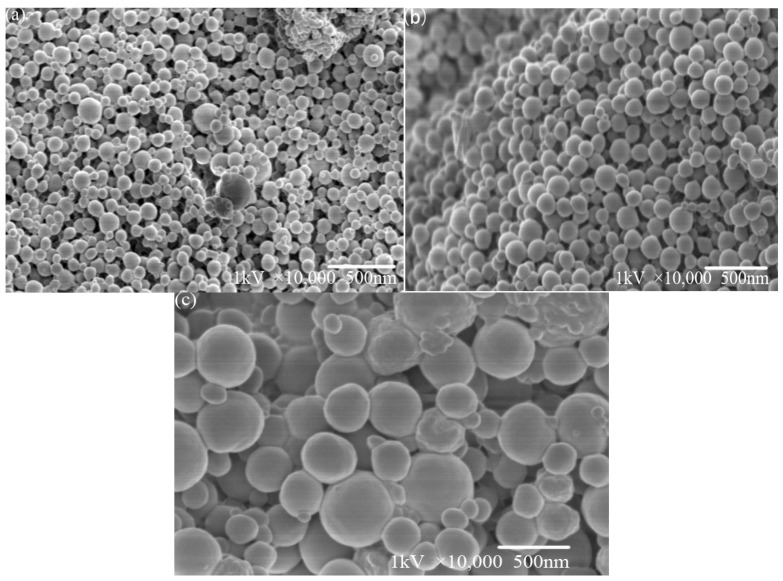
10 k- and 5 k-times SEM images of PLA with different particle sizes: (**a**) 50 nm PLA, (**b**) 100 nm PLA, (**c**) 200 nm PLA.

Figure 5 shows the 10 k- and 5 k-times SEM images of PLA particles with sizes of 50 nm, 100 nm and 200 nm. As can be seen from Figure 5, after the treatment of different particle sizes, PLA particles are mostly spheres or spheroid, most particle surfaces are smooth, and the surface of some spheres is relatively rough. It was found that the particle size of PLA obtained by SEM was larger than seen via DLS, but the particle size distribution was basically stable in the range of 50–200 nm, which was consistent with the results obtained by DLS. As shown in Figure 5a, most of the spheres of PLA particles with a particle size of 50 nm have a particle size of 50–100 nm. The particles are independent of each other and have less adhesion, which is close to the test data of the laser particle size analyzer. The particle size distribution is inhomogeneous, but there are also some larger particles with a size of more than 100 nm. It can be observed in Figure 5b that the synthesized particle size of 100 nm PLA particles, relative to the particle size of 50 nm balls, significantly increased, the size of the small particles less than 100 nm was reduced, and the range of particle sizes is more consistent, most particles being in the range of 100–200 nm. As shown in Figure 5c, most particles in the synthesized PLA with a particle size of 200 nm have a particle size of almost more than 250 nm, and the reduction of small particles under 100 nm is even more pronounced. It can be observed that the particles with smaller particle sizes adhere to each other to form larger agglomerates.

**Figure 6:**
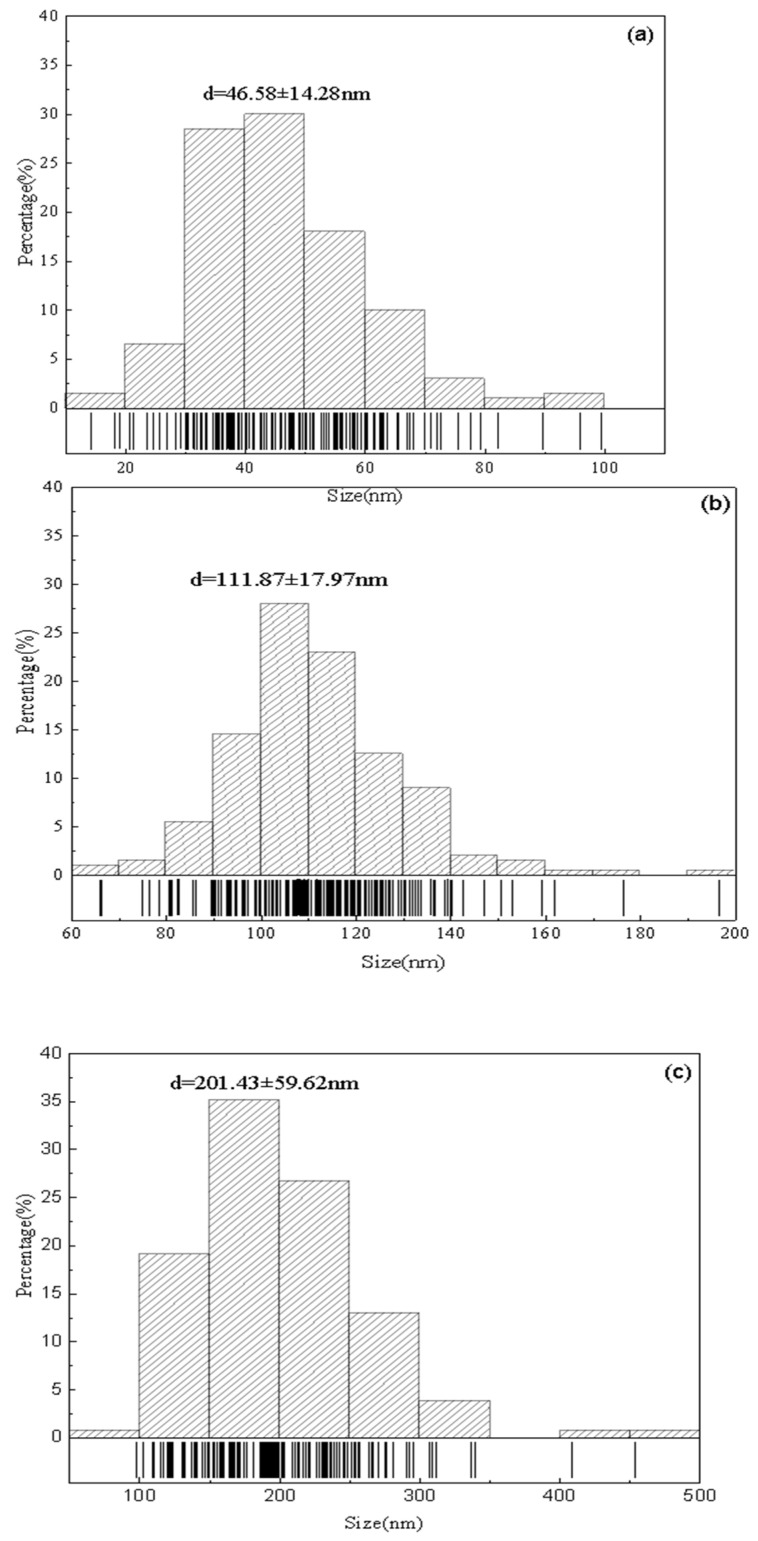
Histogram of particle size distribution of PLA-NPs: (**a**) 50 nm PLA, (**b**) 100 nm PLA, (**c**) 200 nm PLA.

The synthetic parameters were as follows: 50 nm (oil/water ratio 1:7, PLA: 0.01 g, Tween-20: 1 mL); 100 nm (oil/water ratio 1:7, PLA: 0.01 g, Tween-20: 0.3 mL); and 200 nm (oil/water ratio 1:7, PLA: 0.1 g, Tween-20: 0.3 mL). The SEM images were analyzed using Nano Measurer software (https://nano-measurer.software.informer.com/) and the histograms of the particle size distribution of the PLA particles were obtained as shown in Figure 6. The average particle sizes of the particles calculated from the histograms obtained from the SEM images were 46.58 ± 14.28, 111.87 ± 17.97, and 201.43 ± 59.62 nm, which were almost the same as those obtained using the Malvern laser (Malvern Instrument Ltd., Malvern, UK).

### 2.5. FT-IR

**Figure 7:**
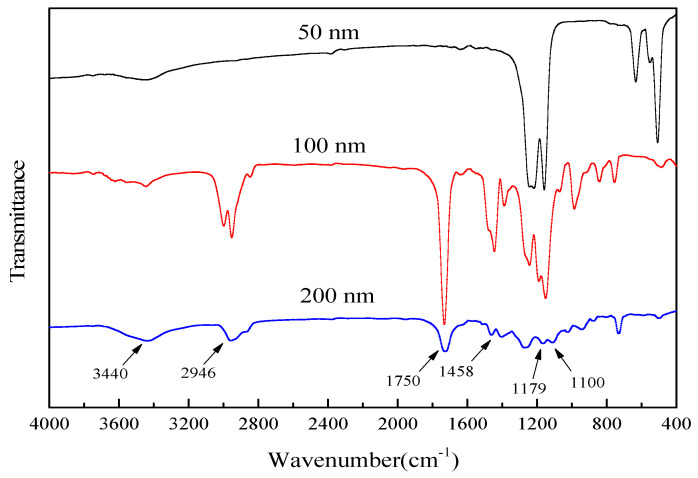
FT-IR plots of PLA particles with different particle sizes.

Synthetic samples were examined using an Agilent Cary 630 Fourier infrared spectrometer (Agilent Technologies, Santa Clara, CA, USA) to determine the main components of the samples. As can be seen from Figure 7, the absorption peak at the wavelength of 400 cm^−1^ is the -OH telescopic vibrational absorption peak [31], which mainly comes from pore water that has not been completely evaporated from the material. The absorption peaks near the wavelengths of 1100, 1179 and 1750 cm^−1^ are the C-O, C-O-C and C=O telescopic vibrational absorption peaks, respectively [32].The absorption peaks near the wavelengths of 1458 and 2946 cm^−1^ are the symmetric and asymmetric telescopic vibrational absorption peaks, respectively, of C-H3 [33], which are mainly derived from the PLA polymer chains of C-H3, C-O-C, C=O and C=O. O-C, C=O, and C-COO groups are also on the PLA polymer chain [13]. All the above functional groups are important functional groups of PLA particles, so the main components of the synthesized particles are all PLA.

## 3. Materials and Methods

### 3.1. Materials

Polylactic acid (PLA $\bar{Mn}$ = 1,000,000 g/mol) was supplied by Shanghai Run Vast Plastic, Shanghai, China. Tween-20 was purchased from Shanghai McLean Biochemical Technology, Shanghai, China. Methylene chloride was purchased from Tianjin Fuyu Fine Chemical, Tianjin, China. The Zetasizer Nano 90 nanoparticle size analyzer was supplied by Malvern Instruments Ltd., Malvern, UK. The ultrasonic cell pulverizer was supplied by Ningbo Xinzhi Bio-Technology Ltd., Ningbo, China. All aqueous solutions were prepared with Milli-Q (Deionized water 18 MΩ) water.

### 3.2. Preparation of PLA-NPs

PLA-NPs were synthesized using the O/W emulsification-solvent evaporation method [34]. The approach mainly involved adding a certain amount of PLA to a defined volume of dichloromethane to form the organic phase, which was placed in a sealed container and then ultrasonicated for 10 min, so that PLA was completely dissolved in dichloromethane. Subsequently, the surfactant was slowly added to ultrapure water using a disposable 1 mL syringe while a glass rod was stirred to dissolve it to form the aqueous solution of a certain concentration, that is, the water phase. Once the aqueous solution was thoroughly mixed, the organic phase was slowly added to the aqueous phase using a disposable rubber-tipped burette, followed by uniform stirring. The organic phase was added to the aqueous phase by slow dropwise addition with a controlled dropwise acceleration of 1 drop per second for a total time of 10 min while maintaining the same room temperature conditions. During the pre-experiment, we found that when the ultrasonication time did not exceed 25 min, the organic phase and the aqueous phase showed an obvious delamination phenomenon, and when the ultrasonication time was 30 min, the organic phase of the aqueous phase showed a homogeneous mixing state. Therefore, ultrasonication was performed using a cell crusher for 30 min (1 s on, 1 s off, 300 w) to mix the aqueous and organic phases evenly. In order to prevent the volatilization of DMC during the sonication process due to the long sonication time, a frozen ice pack was placed under the solution [35]. After completing the sonication process, magnetic stirring (1000 rpm) was conducted overnight at room temperature to ensure the organic solvent could be completely volatilized. The prepared suspensions of PLA-NPs were diluted and the average hydrodynamic diameter (z-Average) and polydispersity index (PDI) were measured by Dynamic Light Scattering (DLS) [9]. PDI is used to describe the molecular weight distribution of polymers. The error bars for PDI and particle size were obtained through three repetitions of the on-line test. When ultrasonic waves were transmitted to the emulsion formed by the mixture of organic phase and aqueous phase through a vortex, the aggregation and dispersion of microbubbles occurs in the medium [18].

### 3.3. Optimization of Particle Size and PDI by One-Factor Experiments

#### 3.3.1. Ultrasonication Time

Throughout the entire system, the total suspension volume remained constant. PLA, organic phase to aqueous phase volume, and Tween-20 were set at 0.01 g, 1:10, and 1 mL, respectively. Ultrasonication time was varied between 20, 25, 30, 35, and 40 min to explore its influence on the preparation of PLA-NPs.

#### 3.3.2. Volume Ratio of Organic Phase to Aqueous Phase

Ultrasonication time, PLA and Tween-20 were set at 30 min, 0.01 g and 1 mL, respectively, and organic phase to aqueous phase volume was set variously to 1:10, 1:7, 1:5, 1:3 and 1:1 in order to examine its influence on the preparation of PLA-NPs.

#### 3.3.3. Surfactant Tween-20

The ultrasonication time, organic phase to aqueous phase volume, and PLA were fixed at 30 min, 1:7, and 0.01 g, respectively. Tween-20 was variously set to 0.25, 0.3, 0.4, 0.5, 0.75, and 1 mL to examine its influence on the preparation of PLA-NPs.

#### 3.3.4. PLA 

The ultrasonication time, organic phase to aqueous phase volume and Tween-20 were set to 30 min, 1:7 and 0.3 mL, respectively, and the range of PLA was set variously to 0.1, 0.2, 0.3, 1, 1.1 and 1.2 g to examine its influence on the preparation of PLA-NPs.

### 3.4. Response Surface Optimization Design

Response Surface Methodology (RSM) is a widely utilized statistical approach for optimizing complex processes mainly through diversified methods [36]. The advantage of RSM is that it can reduce many unnecessary trials when exploring multi-factor interactions, and it is an optimization method that saves time and labor costs [37]. Following the principles of Box-Behnken experimental design [38], comprehensively comparing the effects of single factors on particle size as well as PDI, three factors were screened: organic phase to aqueous phase volume, Tween-20, and PLA. Design Expert software version 11 was employed for data analysis [39], facilitating further exploration of factors influencing nanoparticle size and PDI. The optimized independent variables are shown in Table 4. The table shows organic phase to aqueous phase volume (A), Tween-20 (B), and PLA (C), the chosen response parameters were particle size of PLA and PDI, and each independent variable was allocated both an upper and lower limit.

## 4. Conclusions

In this investigation, the approach for synthesizing PLA-NPs and factors influencing the particle size and PDI of NPs were systematically examined. The results indicated that the application of RSM combined with one-factor control variable methodology compensated for the insufficiency of one-factor analysis in comprehensively assessing the selected parameters. PLA-NPs ranging between 50 and 200 nm were obtained in this study. Experimental findings suggested that the quality of PLA was the primary factor influencing the particle size. PLA-NPs with different particle sizes can be prepared by adjusting PLA concentration. PLA (0.01 g–0.1 g) had a positive correlation with the particle size, which gradually increased as PLA increased from 0.01 g to 0.1 g. Meanwhile, surfactant Tween-20 (0.25 mL–1 mL) exhibited a negative correlation with the particle size. As the surfactant Tween-20 increased from 0.25 mL to 1 mL in the aqueous phase, the particle size of the nanoparticles gradually decreased. The volume ratio of the organic phase to the aqueous phase increased from 1:10 to 1:1, and the particle size first decreased (1:10–1:5) and then increased (1:5–1:1). As the ultrasonication time increased from 20 min to 40 min, the particle size initially increased (from 25 to 30 min) and then decreased (from 30 to 40 min). On the other hand, the amount of Tween-20 was the main effect factor positively correlated with PDI. Therefore, to achieve PDI between 0.2–0.5, Tween-20 was maintained within the range of 0.25 mL–1 mL. When the volume ratio of organic phase to aqueous phase increased from 1:10 to 1:1, PDI first stabilized and then gradually decreased. With the increase of ultrasonication time (20–40 min), PDI tended to be stable after the increase. The effect of PLA on PDI was not significant, and variations in PLA concentration did not lead to significant changes in PDI size. This study offers insights into the preparation of PLA-NPs with diverse particle sizes and stable dispersion systems. Finally, further characterization by SEM revealed that the particle size distribution was basically stable in the range of 50–200 nm, which was consistent with the results obtained by DLS. 

## Figures and Tables

**Table 1:** Experimental factors and response values within the framework of the Box-Behnken design experiment.

Std	Run	Factor			Response	
		A: Organic Phase to Aqueous Phase Volume	B: Tween-20 (mL)	C: PLA (g)	Z-Average (nm)	PDI
6	1	1: 1	0.625	0.01	99.7 ± 0.44	0.187 ± 0.01
14	2	11:20	0.625	0.055	152.7 ± 1.05	0.082 ± 0.02
15	3	11:20	0.625	0.055	170.7 ± 0.45	0.067 ± 0.02
1	4	1:10	0.25	0.055	156.2 ± 0.66	0.063 ± 0.01
3	5	1:10	1	0.055	103.2 ± 1.06	0.36 ± 0.06
7	6	1:10	0.625	0.1	169.9 ± 1.08	0.26 ± 0.00
5	7	1:10	0.625	0.01	101.8 ± 0.68	0.32 ± 0.00
8	8	1:01	0.625	0.1	194.5 ± 2.34	0.05 ± 0.01
2	9	1:01	0.25	0.055	192.7 ± 2.60	0.23 ± 0.01
9	10	11:20	0.25	0.01	143.3 ± 0.26	0.12 ± 0.01
17	11	11:20	0.625	0.055	160.1 ± 0.66	0.12 ± 0.01
16	12	11:20	0.625	0.055	153.5 ± 0.58	0.078 ± 0.01
10	13	11:20	1	0.01	96.5 ± 0.74	0.26 ± 0.01
11	14	11:20	0.25	0.1	207.2 ± 2.15	0.08 ± 0.04

**Table 2:** ANOVA statistical results of fitted quadratic polynomial model for particle size.

Source	SS	D f	Mean Square	F Value	*p*-Value	Significant	R^2^	Adj.R^2^
Model	17,441.36	9	1937.93	14.89	0.0009	**	0.9403	0.8865
A	1371.83	1	1371.83	10.54	0.0141	*		
B	4698.68	1	4698.68	36.09	0.0005	*		
C	10,294.69	1	10,294.69	79.08	<0.0001	**		
AB	21.48	1	21.48	0.165	0.6967	&		
AC	179.16	1	179.16	1.38	0.2791	&		
BC	3.19	1	3.19	0.0245	0.8801	&		
A^2^	434.36	1	434.36	3.34	0.1105	**		
B^2^	1.87	1	1.87	0.0144	0.9079	&		
C^2^	379.74	1	379.74	2.92	0.1314	&		
Std. Dev	11.41							
Mean	151.54							
C.V.%	7.53							
PRESS	10,599.5							

Note: *, significant difference (*p* < 0.05); **, extremely significant difference (*p* < 0.01); &, no significant difference (*p* > 0.05).

**Table 3:** Statistical results of ANOVA for fitting quadratic polynomial model of PDI.

Source	SS	D f	Mean Square	F Value	*p*-Value	Significant	R^2^	Adj.R^2^
Model	0.1478	9	0.0164	11.07	0.0023	**	0.9343	0.8499
A	0.0235	1	0.0235	15.84	0.0053	**		
B	0.0141	1	0.0141	9.51	0.0177	*		
C	0.019	1	0.019	12.83	0.009	**		
AB	0.0461	1	0.0461	31.09	0.0008	**		
AC	0.0015	1	0.0015	1.01	0.3492	&		
BC	0.0038	1	0.0038	2.57	0.1527	&		
A^2^	0.0301	1	0.0301	20.31	0.0028	**		
B^2^	0.0016	1	0.0016	1.1	0.3292	&		
C^2^	0.0052	1	0.0052	3.5	0.1037	&		
Std. Dev	0.0385							
Mean	0.1512							
C.V.%	25.48							
PRESS	0.1392							

Note: *, significant difference (*p* < 0.05); **, extremely significant difference (*p* < 0.01); &, no significant difference (*p* > 0.05).

**Table 4:** Experimental design of three-factor two-level response surface analysis.

Independent Variables		A: Organic Phase to Aqueous Phase Volume	B: Tween-20 (mL)	C: PLA (g)
Level	Low	1:10	0.1	0.01
	Hight	1:1	1	0.1
Dependent variables		particle size (nm)	PDI	

## Data Availability

The data that support the findings of this study are available from the corresponding author upon reasonable request.

## References

[1] Bayda S., Adeel M., Tuccinardi T., Cordani M., Rizzolio F. (2019). The History of Nanoscience and Nanotechnology: From Chemical-Physical Applications to Nanomedicine. Molecules.

[2] He X., Deng H., Hwang H.M. (2019). The current application of nanotechnology in food and agriculture. J. Food Drug Anal..

[3] Abaszadeh F., Ashoub M.H., Khajouie G., Amiri M. (2023). Nanotechnology development in surgical applications: Recent trends and developments. Eur. J. Med. Res..

[4] Teo H.L., Wahab R.A. (2020). Towards an eco-friendly deconstruction of agro-industrial biomass and preparation of renewable cellulose nanomaterials: A review. Int. J. Biol. Macromol..

[5] Ma Y., Xiong L., Lu Y., Zhu W., Zhao H., Yang Y., Mao L., Yang L. (2021). Advanced Inorganic Nitride Nanomaterials for Renewable Energy: A Mini Review of Synthesis Methods. Front. Chem..

[6] Da Silva D., Kaduri M., Poley M., Adir O., Krinsky N., Shainsky-Roitman J., Schroeder A. (2018). Biocompatibility, biodegradation and excretion of polylactic acid (PLA) in medical implants and theranostic systems. Chem. Eng. J..

[7] Ramot Y., Haim-Zada M., Domb A.J., Nyska A. (2016). Biocompatibility and safety of PLA and its copolymers. Adv. Drug Deliv. Rev..

[8] Kaci M., Meziani S., Arab-Tehrany E., Gillet G., Desjardins-Lavisse I., Desobry S. (2014). Emulsification by high frequency ultrasound using piezoelectric transducer: Formation and stability of emulsifier free emulsion. Ultrason. Sonochem..

[9] Ashizawa K. (2019). Nanosize Particle Analysis by Dynamic Light Scattering (DLS). Yakugaku Zasshi.

[10] Sun C., Wang Y., Zhao X. (2018). Research on Preparation Technology of Polylactic Acid Nano-microsphere. J. Agric. Sci. Technol..

[11] Juliano P., Augustin M.A., Xu X.Q., Mawson R., Knoerzer K. (2017). Advances in high frequency ultrasound separation of particulates from biomass. Ultrason. Sonochem..

[12] Freedman R.B., Desmond J.L., Byrne L.J., Heal J.W., Howard M.J., Sanghera N., Walker K.L., Wallis A.K., Wells S.A., Williamson R.A. (2017). Something in the way she moves: The functional significance of flexibility in the multiple roles of protein disulfide isomerase (PDI). Biochim. Biophys. Acta. Proteins. Proteom..

[13] Danaei M., Dehghankhold M., Ataei S., Hasanzadeh Davarani F., Javanmard R., Dokhani A., Khorasani S., Mozafari M.R. (2018). Impact of Particle Size and Polydispersity Index on the Clinical Applications of Lipidic Nanocarrier Systems. Pharmaceutics.

[14] Shao W.Y., He C.Y., Feng Y.L., Chan Y.Q. (2015). Make microspheres through emulsion-solvent evaporation method. J. Funct. Mater..

[15] Park H., Ha D.H., Ha E.S., Kim J.S., Kim M.S., Hwang S.J. (2019). Effect of Stabilizers on Encapsulation Efficiency and Release Behavior of Exenatide-Loaded PLGA Microsphere Prepared by the W/O/W Solvent Evaporation Method. Pharmaceutics.

[16] Jiang W., Zhang H., Xiong X. (2024). Enhancing the Mickering emulsifying capacity of agarose microgels by complexation with microamounts of sorbitan monolaurate (Tween-20). Int. J. Food Eng..

[17] Murakami M., Matsumoto A., Watanabe C., Kurumado Y., Takama M. (2015). Fabrication of porous ethyl cellulose microspheres based on the acetone-glycerin-water ternary system: Controlling porosity via the solvent-removal mode. Drug Discov. Ther..

[18] Marcellus K.A., Bugiel S., Nunnikhoven A., Curran I., Gill S.S. (2024). Polystyrene Nano- and Microplastic Particles Induce an Inflammatory Gene Expression Profile in Rat Neural Stem Cell-Derived Astrocytes In Vitro. Nanomaterials.

[19] Ruiz E., Orozco V.H., Hoyos L.M., Giraldo L.F. (2022). Study of sonication parameters on PLA nanoparticles preparation by simple emulsion-evaporation solvent technique. Eur. Polym. J..

[20] Hasanvand E., Fathi M., Bassiri A. (2018). Production and characterization of vitamin D_3_ loaded starch nanoparticles: Effect of amylose to amylopectin ratio and sonication parameters. J. Food. Sci. Technol..

[21] Ye J., Hua X., Shao X., Yang R. (2024). Acid-induced conformation regulation of peanut polysaccharide and its effect on stability and digestibility of oil-in-water emulsion. J. Sci. Food. Agric..

[22] Shotton E., Davis S.S. (1968). The effect of emulsifier concentration on the rheological properties of acacia emulsions. J. Pharm. Pharmacol..

[23] Tehrani-Bagha A.R., Holmberg K. (2013). Solubilization of Hydrophobic Dyes in Surfactant Solutions. Materials.

[24] Zhang M., Hou J., Xia J., Wu J., You G., Miao L. (2024). The long-term release and particle fracture behaviors of nanoplastics retained in porous media: Effects of surfactants, natural organic matters, antibiotics, and bacteria. Sci. Total. Environ..

[25] Tao C., Huang J., Lu Y., Zou H., He X., Chen Y., Zhong Y. (2014). Development and characterization of GRGDSPC-modified poly (lactide-co-glycolide acid) porous microspheres incorporated with protein-loaded chitosan microspheres for bone tissue engineering. Colloids Surf. B Biointerfaces.

[26] Lakens D. (2013). Calculating and reporting effect sizes to facilitate cumulative science: A practical primer for t-tests and ANOVAs. Front. Psychol..

[27] Chen C.W., Yang H.C. (2019). OPATs: Omnibus P-value association tests. Brief. Bioinform..

[28] Stewart W.H. (1996). Application of response surface methodology and factorial designs to clinical trials for drug combination development. J. Biopharm..

[29] García-Beleño J., Rodríguez de. (2021). San Miguel, E. Integration of Response Surface Methodology (RSM) and Principal Component Analysis (PCA) as an Optimization Tool for Polymer Inclusion Membrane Based-Optodes Designed for Hg (II), Cd (II), and Pb (II). Membranes.

[30] Sun C., Li C., Tan H., Zhang Y. (2019). Synergistic effects of wood fiber and polylactic acid during co-pyrolysis using TG-FTIR-MS and Py-GC/MS. Energy Convers. Manag..

[31] Dinpanah E., Lakouraj M.M., Fooladi E., Hasantabar V. (2014). Synthesis and characterization of a nanostructure conductive copolymer based on polyaniline and polylactic acid as an effective substrate in proteins impedimetric biosensing. RSC Adv..

[32] Joolaei A.A., Makian M., Prakash O., Im S., Kang S., Kim D.H. (2024). Effects of particle size on the pretreatment efficiency and subsequent biogas potential of polylactic acid. Bioresour. Technol..

[33] Liu Y., Liu H., Yuan D., Chen S., Zhu C., Chen K. (2024). The effect of polylactic acid ordering on the long-term corrosion protection capacity of biodegradable magnesium alloys. Int. J. Biol. Macromol..

[34] Rahman Z., Zidan A.S., Habib M.J., Khan M.A. (2010). Understanding the quality of protein loaded PLGA nanoparticles variability by Plackett-Burman design. Int. J. Pharm..

[35] Sánchez-Roa D., Mosquera M.E.G., Cámpora J. (2021). NHC-CDI Betaine Adducts and Their Cationic Derivatives as Catalyst Precursors for Dichloromethane Valorization. J. Org. Chem..

[36] Rajesh C., Rajashekara R., Nagaraju P. (2023). Response Surface Methodology (RSM) modelling for the photocatalytic optimization study of benzophenone removal using CuWO_4_/NiO nanocomposite. J. Environ. Health Sci. Eng..

[37] Agrawal D., Waghe U., Ansari K., Amran M., Gamil Y., Alluqmani A.E., Thakare N. (2024). Optimization of eco-friendly concrete with recycled coarse aggregates and rubber particles as sustainable industrial byproducts for construction practices. Heliyon.

[38] Zhou Y., Yang X., Li Q., Peng Z., Li J., Zhang J. (2023). Optimization of fermentation conditions for surfactin production by B. subtilis YPS–32. BMC Microbiol..

[39] Zaker H., Taymouri S., Mostafavi A. (2022). Formulation and physicochemical characterization of azithromycin-loaded cubosomes. Res. Pharm. Sci..

